# The Humoral Immune Response Against COVID-19 Through Vaccination in Hemodialysis Patients

**DOI:** 10.3390/vaccines13020170

**Published:** 2025-02-10

**Authors:** Ji Young Park, Seong-Ho Choi, Yong Kwan Lim, Jungho Shin, Soie Kwon, Haein Kim, Jin-Won Chung

**Affiliations:** 1 Department of Pediatrics, Korea University Ansan Hospital, Ansan 15355, Republic of Korea; parkjy@kumc.or.kr; 2 Biomedical Research Institute, Chung-Ang University Hospital, Seoul 06973, Republic of Korea; 3 Division of Infectious Diseases, Department of Internal Medicine, Chung-Ang University Hospital, Seoul 06973, Republic of Korea; 4 Department of Laboratory Medicine, Chung-Ang University Hospital, Seoul 06973, Republic of Korea; 5 Division of Nephrology, Department of Internal Medicine, Chung-Ang University Hospital, Seoul 06973, Republic of Korea

**Keywords:** antibodies, neutralizing, renal dialysis, SARS-CoV-2, vaccination

## Abstract

Background: This study investigated the humoral responses to SARS-CoV-2 in hemodialysis (HD) patients. The clearance of molecules in the blood during hemodialysis is influenced by factors such as filter pore size, flow rate, operating pressure, and treatment duration. Chronic kidney disease patients often show low antibody titers for pathogens like pneumococcus, influenza virus, and hepatitis B virus. Methods: In this study, the surrogate virus neutralization test (sVNT) for the wild type (WT) and Omicron variants, as well as spike-specific IgG levels, were measured at two time points (May 2022 and December 2023). Medical records and questionnaires were used to gather participant information. Results: A total of 26 HD patients were enrolled, including 3 on immunosuppressive therapies. A total of 8 patients had COVID-19 during the first sampling, and 19 during the second. The results showed that sVNT levels for WT decreased over time, though positivity remained at 100% during both sampling periods. In contrast, sVNT levels for Omicron increased significantly, with positivity rising from 46.2% to 75.0% (*p* < 0.05). Spike-specific IgG levels also increased, with positivity improving from 96.2% to 100%. Patients on immunosuppressive therapies had significantly lower sVNT levels for both WT and Omicron in the second period (*p* < 0.05), though no significant differences were observed during the first period. Conclusion: HD patients, particularly those on immunosuppressive therapies, showed reduced and declining neutralizing responses over time. A meta-analysis of HD patients seems necessary to determine whether all dialysis patients need COVID-19 booster vaccinations, similar to the hepatitis B vaccine, highlighting the need for targeted vaccination strategies.

## 1. Introduction

After the first infection, the level of antibodies increases over time as B cells are activated. Once the initial antigen exposure subsides, antibody production remains at a low level. However, upon subsequent infections, memory B cells respond much more rapidly and produce antibodies faster than during the initial infection. Vaccination mimics this first infection by triggering a primary immune response. Humoral immune responses to vaccination vary depending on factors such as race, sex, age, and other individual characteristics [1,2]. In South Korea, nationwide COVID-19 vaccinations began on 26 February 2021. According to previous studies, the levels of neutralizing antibodies (NAbs) against COVID-19 showed significant differences by sex after the first dose, but no differences were observed by age among healthy healthcare workers [3]. Additionally, antibody titers following the primary series of COVID-19 vaccination varied depending on the type of vaccine administered (BNT162b2 vs. ChAdOx1 nCoV-19), cellular and humoral immune responses, and the timing of breakthrough infections [4,5].

Patients with chronic kidney disease (CKD) undergoing hemodialysis (HD) were particularly vulnerable during the early stages of the COVID-19 pandemic, experiencing high mortality and hospitalization rates [6,7]. These patients are known to have impaired immune responses and an increased risk of infections [8,9]. Among the various causes of CKD, conditions such as glomerulonephritis or kidney transplantation often require the use of immunosuppressive agents, even after initiating HD [8]. Furthermore, uremia is known to negatively impact immune responses. As a result, HD patients typically show lower rates of seroconversion, reduced antibody titers, and shorter-lasting immune responses following vaccination compared to healthy individuals. The degree and mechanism of immunosuppression depend on the medications being used, leading to variations in the type and level of immune suppression. Given this, vaccination is a crucial strategy for preventing infections in CKD patients [10].

Maintenance HD patients, in particular, often have multiple comorbidities, variability in dialysis methods, and immunological changes caused by uremia, which can be influenced by their dialysis schedules. These factors highlight the need to investigate immune responses to COVID-19 vaccinations in order to establish effective vaccination policies for HD patients after the detailed clinical analyses [8]. In this study, we examined the serial trends of humoral immune responses after COVID-19 booster vaccinations in HD patients, considering key factors such as demographics, the use of immunosuppressive agents, and the administration of vaccines targeting the Omicron variant.

## 2. Materials and Methods

### 2.1. Study Design and Data Collection

In South Korea, Omicron variants became predominant from the 3rd week of January 2022, and the bivalent COVID-19 vaccine was approved on 7 October 2022. This study was a cross-sectional analysis. We enrolled 26 HD patients with CKD, all of whom had received both the primary and booster COVID-19 vaccinations. Among them, 19 patients were diagnosed with COVID-19 once. A total of 3 patients contracted COVID-19 after receiving the primary series of the COVID-19 vaccine, and 16 patients were diagnosed with COVID-19 after receiving the booster vaccination. Of these, three patients contracted COVID-19 after receiving the bivalent vaccine.

Blood samples were collected twice, with a 19-month interval between the first sampling (11–12 May 2022) and the second sampling (6–7 December 2023). The tests for humoral immunity were performed using commercial kits. We also collected data on participants’ birth dates, sex, underlying conditions, prescribed medications, dates of COVID-19 diagnosis at the time of blood sampling, and vaccination dates.

At the time of administering the bivalent vaccine in Korea, a total of four types of bivalent vaccines were supplied. From October 2022, BNT162b2 and mRNA-1273 were distributed as BA.1 vaccines, and from November 2022, they were supplied as BA.4/5 vaccines. During the 2023–2024 season, XBB.1.5 monovalent vaccines of BNT162b2 and mRNA-1273 were distributed.

### 2.2. Assessment of Humoral Immunities

NAb was measured using the GenScript SARS-CoV-2 cPASS surrogate virus neutralization test (sVNT) kit (Genscript Biotech Corporation, Piscataway, NJ, USA) and spike-specific immunoglobulin G (IgG) was measured with the Euroimmun anti-SARS-CoV-2 IgG enzyme-linked immunosorbent assay (ELISA) (Euroimmun, Lübeck, Germany). The sVNT results were semi-quantitative and interpreted based on the percent inhibition of RBD-HRP binding, calculated as (1-optical density (OD) of sample/OD of negative control) × 100. A 30% cutoff is used to differentiate positive from negative samples, calibrated against the gold-standard plaque reduction neutralization test (PRNT) with high-stringency PRNT_90_ (90% plaque reduction) analysis. A percent inhibition of ≥30% indicates the presence of SARS-CoV-2 RBD-interacting antibodies that block the RBD-hACE2 interaction [11].

The test was modified to detect SARS-CoV-2 NAbs against B.1.1.529 Omicron variant RBD by replacing the horseradish peroxidase-conjugated recombinant RBD fragment according to the manufacturer’s specifications. The IgG assay, which uses the S1 domain of the SARS-CoV-2 spike protein, produced results classified as positive (index ≥ 1.1), borderline, or negative (index < 0.8). When borderline IgG results were categorized as negative, the strength of agreement between IgG ELISA and sVNT was higher than when categorized as positive in a previous study [2]. Therefore, the borderline IgG ELISA results were categorized as negative in this study. The Anti-SARS-CoV-2 NCP ELISA IgG assay (Euroimmun, Lübeck, Germany) was performed on patients without a history of COVID-19 infection, following the manufacturer’s instructions. Ratios < 0.8 were considered negative, ≥0.8 to <1.1 were borderline, and ≥1.1 were positive.

### 2.3. Statistical Analysis

All statistical analyses were performed using R version 4.4.1 (http://www.R-project.org/, accessed on 21 January 2025). Continuous variables were expressed as mean values and compared using either the two-sample T-test or Mann–Whitney U test. Statistical significance was set at *p* < 0.05. The correlation between antibody titers and the time interval from booster vaccination or confirmed COVID-19 infection to blood sampling was analyzed by Spearman’s rank correlation analysis and visualized by a locally estimated scatterplot smoothing curve. One vaccination dose or one confirmed COVID-19 infection was considered as one event of exposure.

## 3. Results

Blood samples were collected from 26 HD patients. The mean age of participants was 72.4 ± 10.8 years old and 53.8% (n = 14) were female. Of the total participants, three HD patients (11.5%) were on immunosuppressive therapies due to lupus nephritis and combined rheumatic arthritis. When collecting the 1st blood samples, 2 patients (7.7%) were immunized with only the primary series, and 24 patients (92.3%) were immunized once or twice with the booster vaccine. All patients were immunized with the booster vaccine once or more and 7 patients (26.9%) were immunized with the Omicron variant at the 2nd time of blood sample collection. In the first questionnaire, 8 patients had a history of confirmed COVID-19 infection, and 19 patients had a COVID-19 history in the second questionnaire. Finally, 7 patients were not confirmed with COVID-19 until the 2nd time of blood sample collection. SARS-CoV-2 NCP ELISA IgG was negative in 6 HD patients and borderline in 1 patient. All COVID-19 cases were diagnosed during the Omicron variant-predominant period in South Korea (Table 1).

### 3.1. Antibody Levels at the Time of the First Blood Sample Collection

In May 2022, when the first blood samples were collected, 100% of the samples showed positive sVNT for wild type (WT; sVNT_1), 46.2% were positive sVNT for Omicron (Omicron_1), and 96.2% were positive for IgG (IgG_1). There were no significant differences in the levels of SVNT_1, Omicron_1, and IgG_1 between females and males (94.9% vs. 89.4%, 28.0% vs. 42.2%, and 4.6 vs. 4.2, respectively, all *p* > 0.05; Table 2). Additionally, the values of sVNT_1, Omicron_1, and IgG_1 were not correlated with age (rho = −0.119, −0.210, and −0.175, respectively, all *p* > 0.05). There were no significant differences in sVNT_1, Omicron_1, and IgG_1 levels between patients with and without immunosuppressive therapy (77.5.3% vs. 94.3%, 33.8% vs. 40.9%, and 3.4 vs. 4.6, respectively, all *p* > 0.05). Similarly, there were no differences in SVNT_1, Omicron_1, and IgG_1 between patients with a confirmed history of COVID-19 infection and those without (91.6% vs. 92.7%, 34.2% vs. 34.8%, and 4.6 vs. 4.3, respectively, all *p* > 0.05) (Table 2). Interestingly, SVNT_1 levels were higher in patients with more frequent exposures to COVID-19 (rho = +0.516, *p* = 0.007); however, Omicron_1 and IgG_1 did not differ based on the number of exposures (rho = +0.231 and +0.195, all *p* > 0.05). All these statistical analyses showed the same results even when excluding the three immunocompromised patients (Supplementary Table S1).

**Figure 1:**
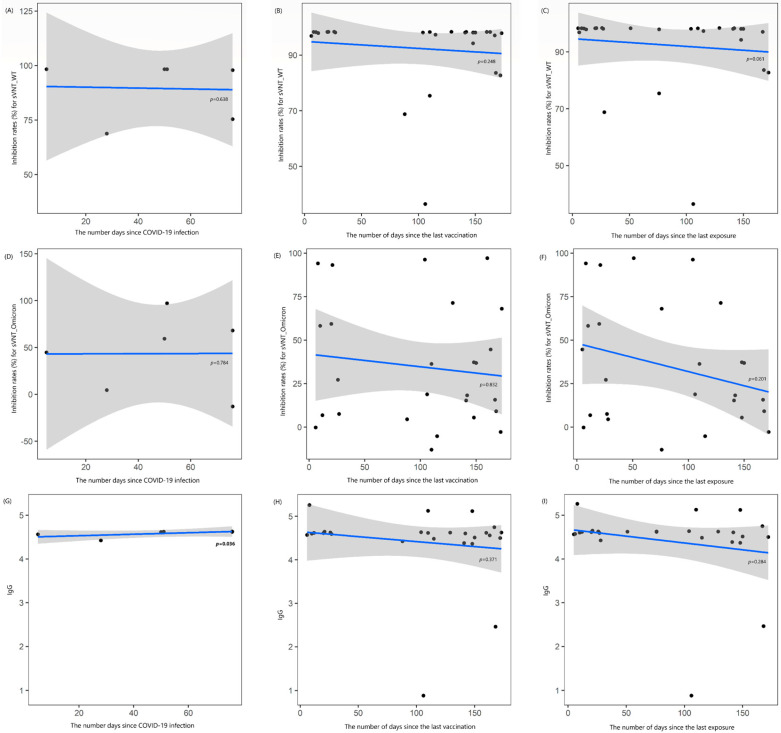
Locally estimated scatterplot smoothing curve based on data from the first blood sample collection, illustrating the relationship of antibody levels as follows: (**A**–**C**) inhibition rates (%) for the surrogate virus neutralization test (sVNT) against the wild-type (WT) strain, (**D**–**F**) inhibition rates (%) against the Omicron variant, and (**G**–**I**) IgG levels. The curves are plotted against the number of days since COVID-19 infection (**A**,**D**,**G**), the last vaccination (**B**,**E**,**H**), and the last exposure to COVID-19 (**C**,**F**,**I**). The blue line represents the regression line, and the shaded gray area indicates the 95% confidence interval.

No correlation was observed between the number of days since COVID-19 infection and the levels of sVNT_1 and Omicron_1 (rho = −0.246 and +0.145, all *p* > 0.05). However, a strong positive correlation was found between the number of days since COVID-19 infection and IgG_1 levels (rho = +0.841, *p* = 0.036). No correlation was found between the number of days since the last vaccination and the levels of sVNT_1, Omicron_1, and IgG_1 (rho = −0.235, −0.044, and −0.183, respectively; all *p* > 0.05), nor was there any correlation between the elapsed times since the last exposure to COVID-19 and these antibody levels (rho = −0.372, −0.259, and −0.218, respectively; all *p* > 0.05) (Figure 1). When these results were further analyzed in HD patients not undergoing immunosuppressive therapy, a longer duration between the last exposure and first blood sampling showed a moderate negative correlation with sVNT_1 (rho = −0.426, *p* = 0.043; Supplementary Figure S1).

### 3.2. Antibody Levels at the Time of the Second Blood Sample Collection

In December 2023, when the second blood samples were collected, 100% of the samples showed positive for sVNT against WT (sVNT_2), 75.0% were positive for sVNT against Omicron (Omicron_2), and 100% were positive for IgG (IgG_2). There were no significant differences in SVNT_2, Omicron_2, and IgG_2 levels between females and males (91.6% vs. 85.9%, 71.5% vs. 59.5%, and 9.0 vs. 8.5, respectively, all *p* > 0.05; Table 2). Additionally, there were no correlations between sVNT_2, Omicron_2, and IgG_2 levels and age (rho = 0.019, −0.034, and 0.183, respectively, all *p* > 0.05). In patients on immunosuppressive therapy, sVNT_2 and Omicron_2 levels were significantly lower compared with patients without immunosuppressive therapy (64.2% vs. 92.2% and 28.9% vs. 70.8%, all *p* < 0.05). However, there were no significant differences in sVNT_2, Omicron_2, and IgG_2 levels between patients who received the vaccines targeting the Omicron variant and those who did not (94.8% vs. 86.8%, 75.7% vs. 62.4%, and 10.2 vs. 8.3, respectively, all *p* > 0.05; Table 2). Similarly, SVNT_2, Omicron_2, and IgG_2 levels were not significantly different between the COVID-19-infected patients and those without a prior infection (88.6% vs. 90.1%, 70.9% vs. 52.6%, and 9.2 vs. 7.6, respectively, all *p* > 0.05) (Table 2). Furthermore, no correlations were found between SVNT_2, Omicron_2, and IgG_2 levels and the frequency of COVID-19 exposures (rho = −0.142, +0.047, and −0.099, respectively, all *p* > 0.05). All statistical analyses yielded the same results even after excluding the three immunocompromised patients (Supplementary Table S1).

**Figure 2:**
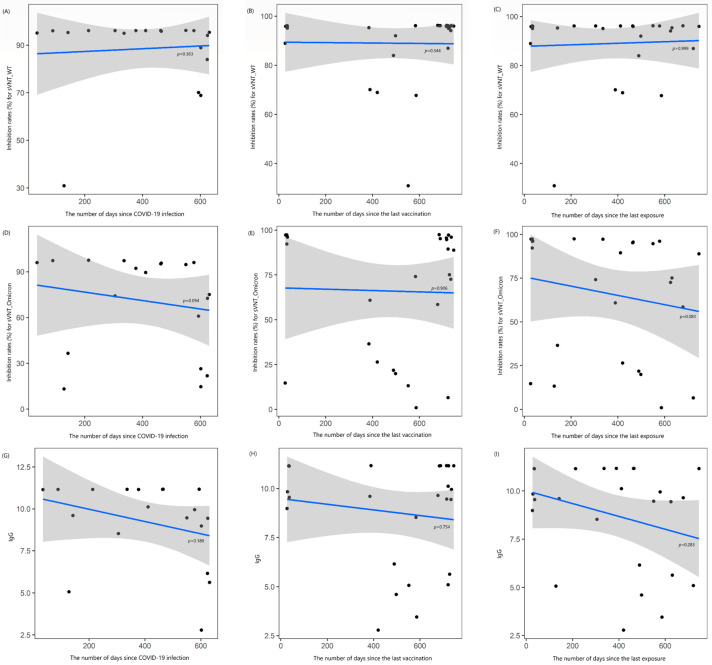
Locally estimated scatterplot smoothing curve based on data from the second blood sample collection, illustrating the relationship of antibody levels as follows: (**A**–**C**) inhibition rates (%) for the surrogate virus neutralization test (sVNT) against the wild-type (WT) strain, (**D**–**F**) inhibition rates (%) against the Omicron variant, and (**G**–**I**) IgG levels. The curves are plotted against the number of days since COVID-19 infection (**A**,**D**,**G**), the last vaccination (**B**,**E**,**H**), and the last exposure to COVID-19 (**C**,**F**,**I**). The blue line represents the regression line, and the shaded gray area indicates the 95% confidence interval.

There were also no correlations between the levels of sVNT_2, Omicron_2, and IgG_2 and the time elapsed since COVID-19 infection (rho = −0.221, −0.395, and −0.321, respectively, all *p* > 0.05), the time elapsed since the last vaccination (rho = +0.193, +0.024, and +0.065, respectively; all *p* > 0.05), or the time elapsed since the last COVID-19 exposure (rho = 0, −0.346, and −0.219, respectively; all *p* > 0.05) (Figure 2). When these results were further analyzed, the duration between the confirmed infection date and the second blood sampling showed a moderate negative correlation with Omicron_2 (rho = −0.574, *p* = 0.016; Supplementary Figure S2).

### 3.3. Changes in Antibody Values over Time Intervals

The inhibition index for sVNT against WT (sVNT_1 to sVNT_2) decreased in 85.7% of HD patients over time. However, the inhibition index for sVNT against Omicron (Omicron_1 to Omicron_2) increased in 78.6% of HD patients, and IgG titers (IgG_1 to IgG_2) increased in 85.7% of patients over time. When analyzed by sex, the difference between sVNT_2 and sVNT_1 decreased by 3.3% in females and 3.5% in males (*p* = 0.860). The difference between Omicron_2 and Omicron_1 was increased by 43.4% in females and 17.3% in males (*p* = 0.129), while the difference between IgG_2 and IgG_1 increased by 4.4 in females and 4.3 in males (*p* = 0.940). The differences in sVNT for WT, sVNT for Omicron, and IgG were not correlated with age (rho = +0.177, +0.201, and +0.051, respectively, all *p* > 0.05).

The difference between sVNT_2 and sVNT_1 decreased by 13.3% in HD patients on immunosuppressive therapy, compared with a 2.1% decrease in patients without immunosuppressive therapy (*p* = 0.018). Conversely, the difference between Omicron_2 and Omicron_1 increased by 37.0% in HD patients without immunosuppressive therapy but decreased by 12.0% in patients on immunosuppressive therapy (*p* = 0.041). The difference between IgG_2 and IgG_1 increased by 2.6 in HD patients on immunosuppressive therapy and by 4.6 in patients without immunosuppressive therapy (*p* = 0.095).

Across all HD patients, the mean differences in sVNT for WT, sVNT for Omicron, and IgG were not different whether patients received the vaccines targeting the Omicron variant or not (all *p* > 0.05). Similarly, there were no significant differences based on a history of COVID-19 infection (all *p* > 0.05). The differences in sVNT for WT, sVNT for Omicron, and IgG were not correlated with the frequency of COVID-19 exposures (rho = +0.062, −0.056, and +0.022, respectively, all *p* > 0.05).

There was no correlation between the differences of sVNT for WT, sVNT for Omicron, and IgG and the time elapsed since COVID-19 infection (rho = +0.007, −0.045, and −0.283, respectively, all *p* > 0.05), the time elapsed since the last vaccination (rho = +0.036, +0.024, and −0.008, respectively; all *p* > 0.05), and the time elapsed since the last COVID-19 exposure (rho = −0.104, −0.153, and −0.316, respectively; all *p* > 0.05).

## 4. Discussion

Over the past four years, COVID-19 has spread worldwide. While many countries have resumed daily life as it was before the pandemic, COVID-19 vaccination is still recommended for the elderly and high-risk patients. In CKD patients undergoing HD, antibodies can be lost through filtration. Moreover, in CKD patients receiving immunosuppressive therapies, weak antibody responses were observed [12,13].

In this study, we analyzed serum samples from HD patients after the initiation of booster vaccinations. Our findings indicated that, over time, the increase in neutralizing antibodies was limited, or even a significant decrease was observed in HD patients on immunosuppressive therapy. After COVID-19 infection, IgG levels showed a strong positive correlation over time at the 1st blood sampling, but this correlation was absent at the 2nd sampling. There was no correlation between antibody levels and the time since the last vaccination or exposure to SARS-CoV-2. At the 1st blood collection, the level of NAb against the WT strain differed based on the number of SARS-CoV-2 exposures, but this was not the case for the Omicron variant and IgG. By the 2nd blood collection, there was no difference in antibody levels based on the number of SARS-CoV-2 exposures.

The 1st blood collection occurred after the predominance of the Omicron variant, and the 2nd blood collection was conducted after the introduction of the bivalent vaccine. By the 2nd blood collection, most participants had been exposed to Omicron variants. The year 2022 was the predominant period for the Omicron variant, with BA.1/1.1 being the dominant sub-lineages in January and February, and BA.2/2.3 being the dominant sub-lineages from March to June, which included the 1st blood collection period [14]. In 2023, the XBB variant became predominant, with HK.3 being the dominant sub-lineage in December, which included the 2nd blood collection period [15].

During the 1st blood collection, IgG levels significantly increased with time following natural COVID-19 infection, but by the 2nd collection, no such correlation was observed. This suggests that most participants had experienced natural infection between two collections and received multiple booster vaccinations. Even though sVNT levels for the Omicron variant and IgG levels increased in participants with a COVID-19 infection history, the differences were not statistically significant. In this study, sVNT_WT showed a decrease in the mean inhibition index at the 2nd blood collection compared to the 1st blood collection (92.37 ± 13.85 vs. 88.98 ± 14.78%), while the sVNT_Omicron value increased in mean inhibition index at the 2nd blood draw compared to the first (34.59 ± 34.70 vs. 65.96 ± 35.18%). This may suggest a gradual waning of the NAb response to the WT strain over time, as well as an increase in natural infections and booster vaccinations to the Omicron variant. However, additional analysis revealed no significant difference in sVNT_Omicron values between individuals infected and not infected with the Omicron variant at the 1st blood sampling (34.16 ± 39.21 vs. 34.78 ± 33.73%, *p* = 0.968), with the same results observed at the second blood sampling (70.88 ± 31.73 vs. 52.61 ± 43.03%, *p* = 0.248). Furthermore, among individuals with a history of COVID-19 infection, there was no significant difference in sVNT_Omicron values between those who received an Omicron variant booster vaccination after natural infection and those who did not (67.35 ± 38.93 vs. 72.14 ± 30.35%, *p* = 0.781). Based on these results, it appears that the NAb response is influenced not by the presence of natural infection or vaccination but rather by biochemical factors such as cytokines or antigen affinity involved in the immune response to the Omicron variant, which differ from those of the wild type.

In patients with autoimmune rheumatic diseases (ARDs) on immunosuppressive therapies, the neutralizing response to the WT strain did not differ between healthcare workers and ARD patients after the primary vaccination series. However, the neutralizing response to the Omicron variant did differ between the two groups after the primary series. There were significant differences in neutralizing response to both WT and Omicron variants after booster vaccination between the two groups [13]. In HD patients, antibody levels significantly increased after booster vaccinations [16], with a more substantial increase observed after the second booster dose compared with the first dose [17]. These increases were correlated with the number of memory B cells [10,18]. Additionally, levels of interleukin (IL)-1β, IL-4 (a Th2-related cytokine), and IL-17 were significantly higher in the HD group than in healthy participants three weeks after booster vaccination [19,20]. IL-1β plays a crucial role in activating the innate immune system, and it triggers IL-4, which enhances the acquired immune response [19,20]. IL-17 is associated with antigen-specific T-cell responses and antibody production [21]. Therefore, the booster vaccination effectively activated innate immune memory in HD patients, with IL-1β likely inducing IL-4. This, in turn, boosted antibody production and increased memory B cells with the involvement of IL-17. Furthermore, IL-2 levels (a Th1-related cytokine) were also elevated in the HD group, indicating that the booster vaccination partially promoted cellular immune responses [10]. Immunomodulators regulate the immune system through various mechanisms. Among them, immunosuppressants can induce non-specific immunosuppression, such as steroids, but most drugs have specific immunomodulating sites of action [22]. For example, commonly used drugs such as azathioprine and mycophenolate mofetil target T cells and B cells, while tacrolimus, a representative immunosuppressive agent, acts on T cells to suppress the immune response [23]. These drugs are widely used in patients with CKD caused by autoimmune diseases. The responses of T cells and B cells play a crucial role in the cellular and humoral immune responses following vaccination and natural infection, making their cell function highly important.

This study has several limitations. First, only 26 patients were enrolled. Although 42 HD patients were initially enrolled during the 1st blood sampling, 16 withdrew their consent by the time of the 2nd blood collection. Nonetheless, the findings remain meaningful as studies on HD patients are limited, and obtaining sufficient participants is challenging. Second, we did not analyze the cellular immune responses in these patients due to delays in acquiring the necessary testing kits. Lastly, most HD patients received a combination of different vaccines, such as ChAdOx1 nCoV-19, BNT162b2, or mRNA-1273, which made subgroup analysis based on vaccine type impossible. While 11 HD patients received two doses of BNT162b2 for their primary series, 9 patients received two doses of ChAdOx1 nCoV-19 and 6 received a mix of ChAdOx1 nCoV-19 and BNT162b2 for their primary series. All HD patients received mRNA booster vaccines (BNT162b2 or mRNA-1273).

As of August 2024, the number of COVID-19 patients is increasing in South Korea. However, no reports of critically ill patients have emerged. At the beginning of the COVID-19 pandemic, HD patients were classified as high risk due to the lack of data on vaccine efficacy in this population. Consequently, they were prioritized for booster vaccinations. Now, more studies have been conducted globally, and these data are essential for refining vaccination policies for this population. Although our study included a small sample size, it is one of the few that analyzed antibody titers and examined factors influencing these titers in HD patients. Our data suggested that while vaccination is critical for HD patients on immunosuppressive therapies, it may not be necessary for all HD patients. When examining studies conducted at other medical centers in Korea that investigated the humoral response in HD patients, one study reported no patients on immunosuppressive therapy [24], while another study included five patients taking steroids or mycophenolate mofetil [25]. As such, the number of cases involving HD patients on immunosuppressive therapy, which is the focus of this study, remains limited. Therefore, to determine whether HD patients as a whole are still a high-risk group for COVID-19, or if only those on immunosuppressive therapy are high-risk, as suggested by the results of this study, further multi-center, multinational studies or meta-analyses are warranted. The routine recommendation of COVID-19 booster vaccination for HD patients, similar to that for hepatitis B and influenza virus, may not be strictly necessary.

## 5. Conclusions

Antibody neutralization exhibited a lower and decreasing trend in HD patients receiving immunosuppressive therapy, highlighting the importance of immunization for this subgroup. Additionally, meta-analyses are needed to investigate both cellular and humoral immune responses to SARS-CoV-2 in HD patients undergoing immunosuppressive therapies.

## Figures and Tables

**Table 1:** Demographics of participants enrolled in this study.

	1st Period (2022)	2nd Period (2023)
	N (%)	
Sex		
Female	14 (53.8)	
Male	12 (46.2)	
Mean age (±SD ^1^, years)	72.4 ± 10.8	
On immunosuppressive therapy	3 (11.5)	
Vaccination status		
Only primary series	2 (7.7)	-
Booster, once	17 (65.4)	13 (50.0)
Booster, twice	7 (26.9)	7 (26.9)
Booster, thrice	-	2 (7.7)
Booster, four times	-	4 (15.4)
Booster with Omicron variant	-	7 (26.9)
BNT162b2	-	6 (85.7)
BA.1	-	1
BA.4/5	-	3
XBB.1.5	-	5
mRNA-1273	-	1 (14.3)
BA.1	-	1
XBB.1.5	-	1
Confirmed COVID-19 history		
Yes	8 (30.8)	19 (73.1)
None	18 (69.2)	7 (26.9)

^1^ Abbreviation: SD, standard deviation.

**Table 2:** Antibody titers from the first and second blood samples by the participants’ demographics.

	sVNT_1	sVNT_2	Omicron_1	Omicron_2	IgG_1	IgG_2
	%					
Sex						
Female	94.9	91.6	28.0	71.5	4.6	9.0
Male	89.4	85.9	42.2	59.5	4.2	8.5
*p*-value	0.423	0.587	0.231	0.681	0.877	0.625
On immunosuppressive therapy						
Yes	77.5	64.2	33.8	28.9	3.4	6.0
None	94.3	92.2	40.9	70.8	4.6	9.1
*p*-value	0.572	0.024	0.705	0.045	0.602	0.054
Bivalent vaccination status						
Booster	-	94.8	-	75.7	-	10.2
No booster	-	86.8	-	62.4	-	8.3
*p*-value	-	0.727	-	0.162	-	0.470
History of COVID-19 infection						
1st sampling period						
Yes	91.6	-	34.2	-	4.6	-
None	92.7	-	34.8	-	4.3	-
*p*-value	>0.999	-	0.892	-	0.637	-
2nd sampling period						
Yes	-	88.6	-	70.9	-	9.2
None	-	90.1	-	52.6	-	7.6
*p*-value	-	0.793	-	0.340	-	0.214

## Data Availability

The raw data supporting the conclusions of this article, excluding personal information, will be made available by the authors upon request.

## Supplementary Materials

The following supporting information can be downloaded at: https://www.mdpi.com/article/10.3390/vaccines13020170/s1, Supplementary Table S1: Antibody titers from the first and second blood samples by the participants’ demographics except ongoing immunosuppressive therapy; Supplementary Figure S1. Locally estimated scatterplot smoothing curve based on data from the first blood sample collection, illustrating the relationship of antibody levels; Supplementary Figure S2. Locally estimated scatterplot smoothing curve based on data from the second blood sample collection, illustrating the relationship of antibody levels.

## Abbreviations

The following abbreviations are used in this manuscript:

CKD	Chronic kidney diseases
HD	Hemodialysis
NAb	Neutralizing antibody
sVNT	Surrogate virus neutralization test
IgG	Spike-specific immunoglobulin G
ELISA	Enzyme-linked immunosorbent assay
OD	Optical density
PRNT	Plaque reduction neutralization test
ARD	Autoimmune rheumatic diseases
