# Supplementary material for: The Humoral Immune Response Against COVID-19 Through Vaccination in Hemodialysis Patients
Source: Vaccines (Basel). 2025 Feb 10;13(2):170. doi: 10.3390/vaccines13020170 (PMC11862019; doi:10.3390/vaccines13020170)
Supplement: Supplementary file 1 [file vaccines-13-00170-s001.zip › vaccines-3411282-supplementary.pdf]

Supplementary Table S1. Antibody titers from the first and second blood samples by the participants' demographics except ongoing immunosuppressive therapy

	sVNT_1	sVNT_2	Omicron_1	Omicron_2	IgG_1	IgG_2
	%					
Sex						
Female	94.4	93.3	24.0	77.2	4.6	9.5
Male	92.2	90.9	44.4	63.8	4.5	8.8
p value	0.961	0.474	0.154	0.343	0.576	0.537
Bivalent vaccination status						
Booster	-	94.8	-	75.7	-	10.2
No booster	-	91.1	-	68.6	-	8.7
p value	-	0.154	-	0.640	-	0.070
History of COVID-19 infection						
1st sampling period						
Yes	90.6	-	25.2	-	4.6	-
None	95.9	-	37.5	-	4.5	-
p value	0.325	-	0.433	-	0.928	-
2nd sampling period						
Yes	-	91.6	-	74.2	-	9.4
None	-	95.4	-	61.2	-	8.3
p value	-	0.569	-	0.415	-	0.358

*Abbreviation: sVNT, surrogate virus neutralization test; sVNT_1, sVNT for wild type when sampling the first blood; sVNT_2, sVNT for wild type when sampling the second blood; Omicron_1, sVNT for Omicron variant when sampling the first blood; Omicron_2, sVNT for Omicron variant when sampling the second blood; IgG_1, IgG when sampling the first blood, IgG_2, IgG when sampling the second blood

Supplementary Figure S1. Locally estimated scatterplot smoothing curve based on data from the first blood sample collection, illustrating the relationship of antibody levels

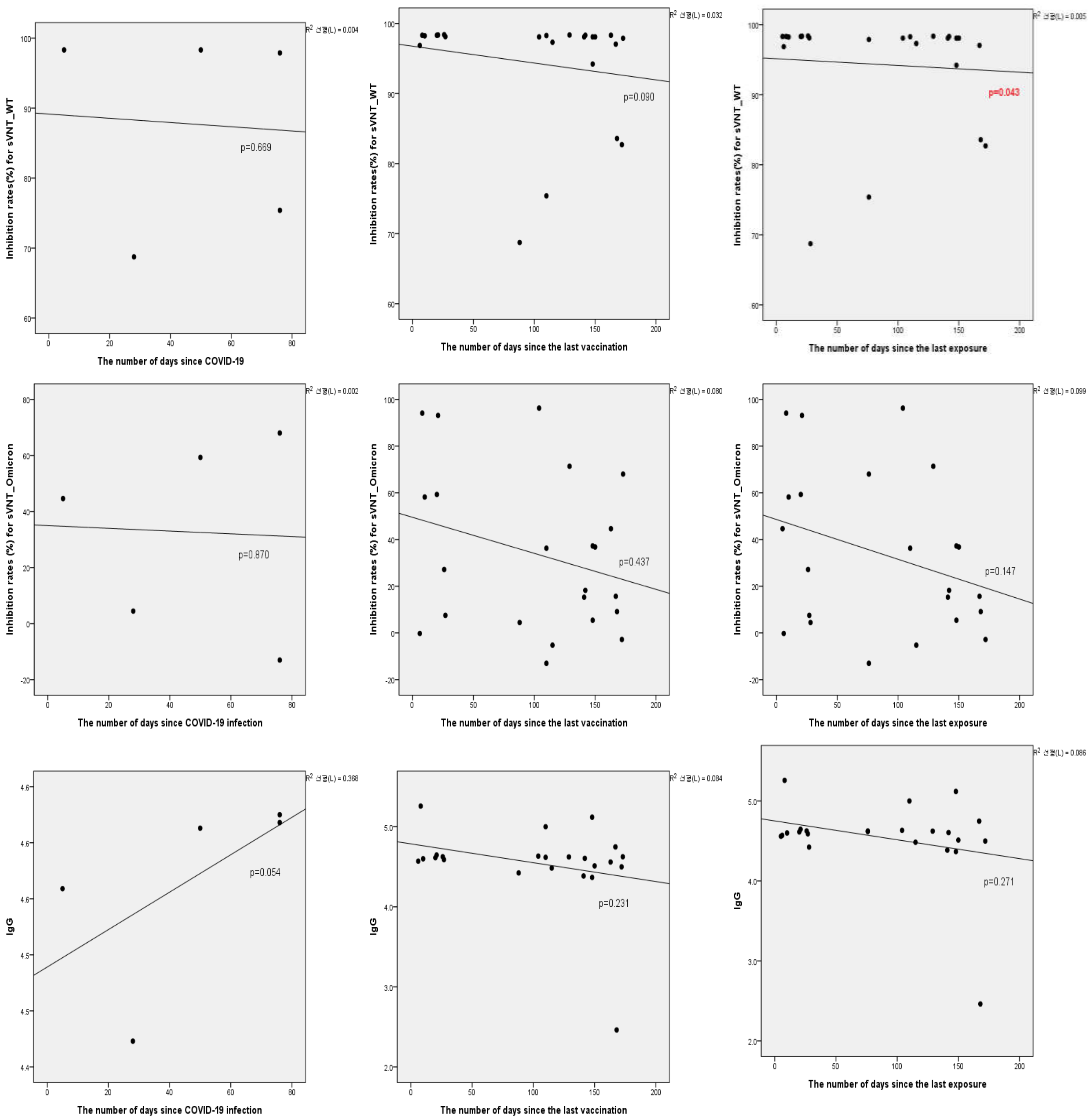

*Abbreviation: sVNT, surrogate virus neutralization test; WT, wild type

Supplementary Figure S2. Locally estimated scatterplot smoothing curve based on data from the second blood sample collection, illustrating the relationship of antibody levels

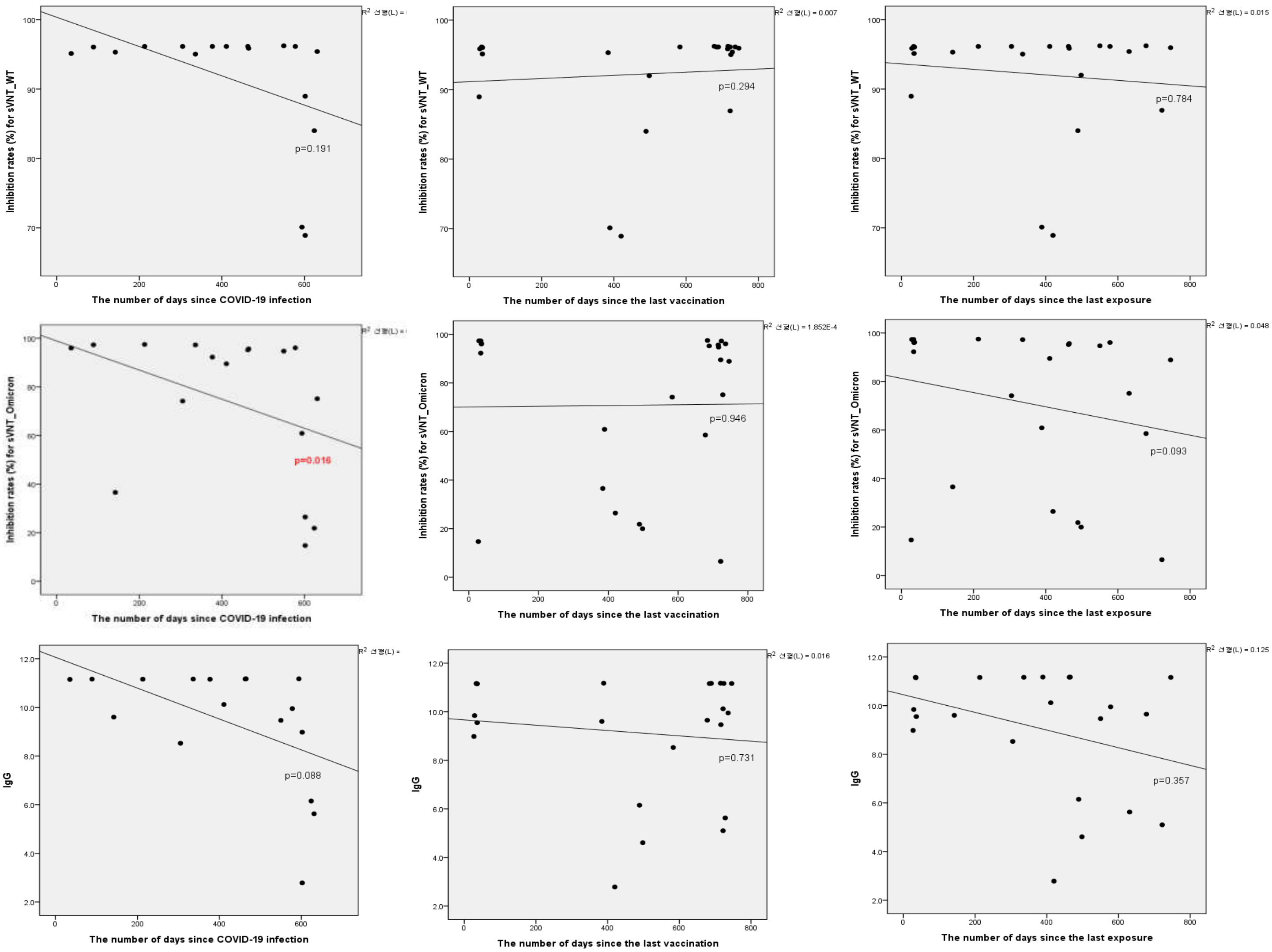

*Abbreviation: sVNT, surrogate virus neutralization test; WT, wild type
